# REsearch into implementation STrategies to support patients of different ORigins and language background in a variety of European primary care settings (RESTORE): study protocol

**DOI:** 10.1186/1748-5908-7-111

**Published:** 2012-11-20

**Authors:** Anne MacFarlane, Catherine O’Donnell, Frances Mair, Mary O’Reilly-de Brún, Tomas de Brún, Wolfgang Spiegel, Maria van den Muijsenbergh, Evelyn van Weel-Baumgarten, Christos Lionis, Nicola Burns, Katja Gravenhorst, Christine Princz, Erik Teunissen, Francine van den Driessen Mareeuw, Aristoula Saridaki, Maria Papadakaki, Maria Vlahadi, Christopher Dowrick

**Affiliations:** 1 Graduate Entry Medical School, University of Limerick, Limerick, Ireland; 2 General Practice and Primary Care, Centre for Population and Health Sciences, College of Medical, Veterinary and Life Sciences, University of Glasgow, 1 Horselethill Road, Glasgow, Scotland, G12 9LX, UK; 3 Discipline of General Practice, School of Medicine No. 1 Distillery Road, National University of Ireland, Galway, Ireland; 4 Department of General Practice, Centre for Public Health, Medical University of Vienna, Kinderspitalgasse 15/1st floor, Vienna, 1090, Austria; 5 Department of Primary and Community Care, 161 ELG, Radboud University Nijmegen Medical Centre, PO Box 9101, Nijmegen, 6500 HB, The Netherlands; 6 Faculty of Medicine, University of Crete, Heraklion, Greece; 7 Institute of Psychology, Health and Society, University of Liverpool, Waterhouse Building, Block B, 1st Floor, 1-5 Brownlow Street, Liverpool, L69 3GL, UK

## Abstract

**Background:**

The implementation of guidelines and training initiatives to support communication in cross-cultural primary care consultations is *ad hoc* across a range of international settings with negative consequences particularly for migrants. This situation reflects a well-documented translational gap between evidence and practice and is part of the wider problem of implementing guidelines and the broader range of professional educational and quality interventions in routine practice. In this paper, we describe our use of a contemporary social theory, Normalization Process Theory and participatory research methodology—Participatory Learning and Action—to investigate and support implementation of such guidelines and training initiatives in routine practice.

**Methods:**

This is a qualitative case study, using multiple primary care sites across Europe. Purposive and maximum variation sampling approaches will be used to identify and recruit stakeholders—migrant service users, general practitioners, primary care nurses, practice managers and administrative staff, interpreters, cultural mediators, service planners, and policy makers. We are conducting a mapping exercise to identify relevant guidelines and training initiatives. We will then initiate a PLA-brokered dialogue with stakeholders around Normalization Process Theory’s four constructs—coherence, cognitive participation, collective action, and reflexive monitoring. Through this, we will enable stakeholders in each setting to select a single guideline or training initiative for implementation in their local setting. We will prospectively investigate and support the implementation journeys for the five selected interventions. Data will be generated using a Participatory Learning and Action approach to interviews and focus groups. Data analysis will follow the principles of thematic analysis, will occur in iterative cycles throughout the project and will involve participatory co-analysis with key stakeholders to enhance the authenticity and veracity of findings.

**Discussion:**

This research employs a unique combination of Normalization Process Theory and Participatory Learning and Action, which will provide a novel approach to the analysis of implementation journeys. The findings will advance knowledge in the field of implementation science because we are using and testing theoretical and methodological approaches so that we can critically appraise their scope to mediate barriers and improve the implementation processes.

## Background

Access to healthcare is a fundamental human right [1], which is promoted within European policy [2]. However, a recent review by Scheppers *et al*. [3] found that migrants’ lack of local language skills is one of the major factors that prohibit the use of health services. Cultural differences, within which gender and ethnicity issues are embedded, can also act as barriers to healthcare [4]. Professional and trained interpreters can enhance understanding and information exchange in cross-cultural general practice consultations [5-8] and evidence-based guidelines and training initiatives to encourage their use in practice are available in European settings [9] and *e.g.*, http://www.nuigalway.ie/general_practice/news.html and http://knmg.artsennet.nl/Publicaties/KNMGpublicatie/KNMGstandpunt-Tolken-in-de-zorg-2011.htm.

However, despite the availability of such guidelines and training initiatives, the use of professional trained interpreters in primary care settings is *ad hoc* and less than ideal across settings and over time [5,10-13]. Migrants and health professionals across European primary care settings generally rely on family members and friends as informal interpreters and cultural mediators [11,12] with a range of negative consequences for their health processes and outcomes, including a lack of understanding about the consultation outcome, poorer compliance with medication, and a lower satisfaction with the consultation [3-5,14]. Our study is seeking ways to address this problem and to enhance uptake of the available guidelines and training initiatives in routine practice.

Poor implementation of available guidelines and training initiatives in the context outlined above reflects a well-recognized and documented problem in the field of implementation science—the translational gap between evidence and practice [15-17]. Surprisingly, apart from some studies that have examined the low uptake of trained professionals to support communication in cross-cultural general practice consultations [13,18], there has been little translational research about the implementation of guidelines or training initiatives to support communication in cross-cultural primary care consultations. Of course, a considerable amount is known about the implementation of clinical guidelines and the broader range of professional educational and quality interventions more generally. Taking the example of clinical guidelines, it is well established that mere dissemination of guidelines is not sufficient and does not guarantee implementation in routine practice; research shows that combined implementation strategies with many different aspects are more effective than single implementation strategies [16,17]. Both the healthcare and management literature have sought to explain why implementation of guidelines is far from straightforward, with existing reviews offering important syntheses of knowledge about the factors that influence the implementation of guidelines [19-21]. In particular, it has been suggested that ‘evidence’ is not unambiguous, but rather is often contested, being reframed in different contexts, which can involve ‘power struggles’ between professional groups [22,23].

To advance knowledge about implementation more broadly, there have been calls to incorporate and investigate the utility of theoretical frameworks into implementation research [22,24-26]. There are a range of theories and conceptual approaches available and in use in the field [19,27-30]. However, in their review of the use of theory in implementation research, Davies *et al*. [31] concluded that we need greater use of explicit theory to understand barriers, design interventions, and explore mediating pathways and moderators to enhance implementation processes. Similarly, Helfrich *et al*. [30] observed that there are few studies which use theory prospectively and which evaluate whether or not the perceived strengths of using theory are borne out, *i.e.*, whether or not theory is found to enhance successful implementation. Therefore, the focus in this study is on using theory as something more than an explanatory heuristic device to understand implementation journeys. This is about using theory to intervene and shape implementation journeys. A key question is whether theoretical knowledge about implementation processes can be used to mediate rather than simply explain barriers to successful implementation [26]. There are empirical challenges to consider in this regard, for example how to operationalize theoretical knowledge so that it can be enacted in fieldwork among a host of individuals, teams, and systems in real space and time in healthcare settings.

In considering these issues, we have looked to the wider literature on barriers and facilitators to the implementation of innovations (be they guidelines, training initiatives, or services) that have been published in the health literature [22,32], but also the organizational and management literature [33,34], to identify what lessons can be learned. Greenhalgh [22], in her influential review of the diffusion of innovations in service organizations, highlighted a number of issues for attention in future implementation research, which included suggestions that: it should be process oriented so that attention is focused on the features that underpin success or failure of implementation rather than on, for example, the content of the guideline being implemented; implementation processes should be examined in a consistent fashion across different contexts or settings; implementation research should be meticulously detailed to allow comparisons across contexts; and it should be participatory, engaging ‘on the ground’ practitioners as partners in the research process.

Clearly, in order to address such recommendations, there is a need to explore the use of innovative methodological strategies in implementation research [35,36]. The idea that implementation should be examined using participatory methods would seem to have particular potential because we know that participatory action research is a collaborative reflective approach that can be used to explore the complex nature of organizational settings [37], and that can lead to important individual and organizational transformations [38,39]. However, to our knowledge, theories of implementation science have not been partnered with participatory research approaches before, and therefore this represents a novel approach to the analysis of implementation processes, but one which thorough review has suggested as worthy of further investigation [22].

The focus of the research agenda for RESTORE (**RE**search into implementation **ST**rategies to support patients of different **OR**igins and language background in a variety of **E**uropean primary care settings) is on conducting primary care research across a number of diverse European settings in a consistent and systematic fashion, that will investigate and support the implementation of guidelines and training initiatives that have been designed to support communication in cross-cultural general practice consultations. We are applying theory prospectively to our implementation research [24,30] and we will employ innovative methods that are action oriented [35,36]. Our attention is on a partnership of contemporary social theory—Normalization Process Theory (NPT) [29]—and Participatory Learning and Action methodology (PLA) [40], which we outline below along with our rationale for their combined use.

### NPT and PLA

NPT is a contemporary sociological theory that explains the dynamics of implementing, embedding, and integrating new technologies or complex interventions into routine settings [29]. Unlike other theories [27,28] it is concerned with the work that people do to operationalize complex interventions and new technologies into their daily routine. It is unique in that it has been derived from empirical generalizations developed within studies of implementation and integration processes in mainstream health [41].

**Figure 1:**
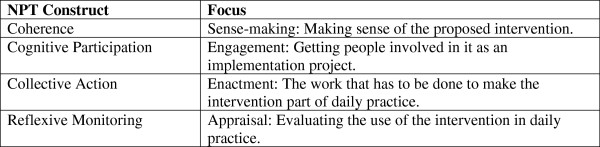
An overview of NPT constructs.

There are four components in NPT: coherence (sense-making), cognitive participation (engagement), collective action (enactment), and reflective monitoring (appraisal) (Figure 1). Each of these has subcomponents that can be used by researchers as sensitizing concepts in implementation research, or to generate propositions for testing in empirical studies [29].

The NPT is not a rigid model but is designed to emphasize the realities of implementation work in real time and space and the inter-relationships between different kinds of implementation work. NPT offers an important theoretical framework, which serves as a heuristic device with which to ‘think through’ implementation issues. NPT has been used to ‘alert’ researchers and stakeholders involved in implementation work to a range of macro-, meso- and micro-level issues that are likely to be encountered [42]. NPT has also been successfully used to retrospectively analyze emergent practices and processes as experienced by those directly involved in implementation projects [43-46]. When applied prospectively, NPT should raise awareness about levers and barriers to successful implementation and, arguably, this awareness could be used to minimize barriers and maximize levers with a view to improving the chances of successful implementation and normalization. This specific prospective use of NPT has not yet been investigated and merits exploration.

However, NPT is not a methodology, and in RESTORE we will combine NPT with a PLA research methodology [40] which, like other action-oriented approaches [47], has the capacity to add a reflexive, problem-solving dimension to the investigation of, and support for, implementation processes. PLA is a practical, adaptive research strategy that enables diverse groups and individuals to learn, work, and act together in a co-operative manner, to focus on issues of joint concern, identify challenges, and generate positive responses in a collaborative and democratic manner [40]. PLA is highly relevant for the field of implementation science because it is a pragmatic multi-perspectival research methodology. There are some recent examples of its use in this field [37,38,48].

**Figure 2:**
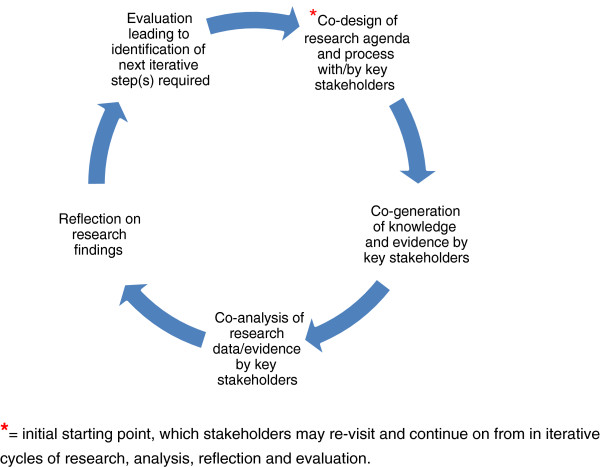
Participatory learning and action research methodology: cycles of research, co-analysis, reflection and evaluation over time.

The iterative and organic nature of PLA encourages diverse stakeholders to engage in cycles of research, co-analysis, reflection, and evaluation over time (Figure 2). This process enables stakeholders to address issues of joint concern creatively in order to arrive at positive strategies to achieve goals, implement agreed actions, and influence national or local policy [49]. A PLA-brokered dialogue refers to the process through which key stakeholder groups are encouraged to listen to, and learn from, each other’s knowledge and perspectives. Trust, rapport, and mutual respect builds up in the early stages of engagement, and this supports the ongoing cycles of work (*i.e.*, research, co-analysis, reflection, and evaluation) [50].

In PLA-brokered dialogues, we aim to create a level playing field, where all perspectives count, and the knowledges embedded in them are shared and enhanced ‘around the stakeholder table’. Taking this approach to research is particularly appropriate when working with stakeholder groups where asymmetrical power relations may exist or very different ideational systems may be in operation—it provides significantly more scope for less powerful groups to speak from their perspective and have their voices heard.

In PLA-brokered dialogues, we pay particular attention to what can be described as emic perspectives. Notwithstanding the complexity of positionality in research [51] and taking into account contemporary notions of culture as a dynamic and fluid reality [52], emic perspectives are understood to provide insight into the full range of lived experiences that ‘insiders’ possess in any specific local and cultural setting. In PLA, we seek to engage stakeholders in the co-design of the research process, the co-generation of materials for data generation, and co-analysis of that data so that their ‘insider’ language and categories of analysis are brought to bear on the research. This is in contrast with more etic approaches to research whereby research processes are planned by researchers only, without such engagement by stakeholders [40,49].

To this end, PLA involves a series of important reversals: from assuming knowledge to exploring and exchanging complex ‘knowledges’; from hierarchical relationships amongst stakeholders to reciprocal and mutually empowering relationships; and from perceptions of stakeholders as passive beneficiaries to acknowledgement of stakeholders as active partners and collaborators who benefit differentially from research outcomes. These reversals require a specific attitudinal disposition on the part of the researcher/practitioner towards stakeholders. This attitudinal disposition is a core and defining feature of the use of PLA in practice.

### Combining NPT with PLA

In our research, we are combining NPT and PLA and exploring whether, if taken together, NPT and PLA can enhance knowledge and action for implementation processes. While NPT has been derived from the empirical generalizations developed within studies of implementation and integration processes in mainstream healthcare, it does represent an etic perspective on implementation processes because it presents an account of implementation work that is not derived from the specific local and cultural setting that a new and unique group of implementers inhabit. Therefore, it is important that the NPT constructs are included in the PLA dialogue but that these constructs are also tested by paying careful attention to emic perspectives, which may be important and which may fall outside the conceptual framework of NPT. Importantly, our research aims to explore whether this novel combination of NPT and PLA to investigate and support the selection of clinical guidelines, and co-design of implementation projects to enhance communication in cross-cultural general practice consultations, will increase the chances of successful implementation and normalization of the selected guidance. By undertaking this work in a systematic and meticulously documented fashion across different European contexts, we will be able to detail the processes by which the selected interventions are adopted and embedded into practice and, importantly, explain why they may or may not be sustained and normalized in routine practice.

The specific research objectives for RESTORE are to determine:

1. What guidelines and training initiatives to support communication in cross-cultural primary care consultations are currently available in the selected European primary care settings?

2. How are the identified guidelines and training initiatives translated into practice in these primary care settings? What are the processes of implementation ‘on the ground’ in routine practice?

3. What is the capacity of these primary care settings in different countries, and therefore different organizational contexts, to incorporate the identified guidelines and training initiatives into their current organizational arrangement?

4. Is the implementation work for the identified guidelines and training initiatives sustainable*—*leading to normalized use of these technologies in routine clinical practice in primary care?

5. What are the benefits, if any, of using NPT and PLA to investigate and support implementation processes?

### Context and method

RESTORE is being conducted in six European settings with fieldwork on implementation journeys in five of these settings (Ireland, England, the Netherlands, Austria, and Greece) and a focus on health policy analysis in the sixth (Scotland). Taken together, RESTORE countries: host a diverse range of migrants from around the world; have different histories of inward migration in terms of colonial ties and migration rates; and have different capabilities and resources in primary care to respond to the complex health and social care needs of migrants [53].

Therefore, this research offers scope for important international comparisons across diverse settings.

We have ethical approval from our respective national committees in the five fieldwork settings: The Irish College of General Practitioners; Liverpool Local Research Ethics Committee; Ethics Committee of Medical University of Vienna; Research Ethics Committee Radboud University Nijmegen Medical Centre; Ethical Committee at the University Hospital of Heraklion, Crete and National Drug Organization (EOF) (Additional files 1, 2, 3, 4, 5 and 6.

The method involves a mapping exercise of guidelines and training initiatives that are designed to support communication in cross-cultural primary care consultations in each fieldwork setting, and a qualitative case study analysis of the implementation journeys of selected interventions from that mapping exercise.

### Mapping exercise

We will take a systematic approach to the mapping exercise, which will include: literature review of relevant peer-reviewed published research from each research setting; literature review of grey literature (professional and policy documents that describe guidelines and training initiatives) in each setting; and qualitative interviews with key informants in each setting (*e.g.* professional bodies, policy makers) using snowball and network sampling methods to identify and recruit an appropriate purposeful sample of informants.

We will develop a protocol for the mapping exercise so that there is consistency in the process across partner countries. The protocol will provide guidance on the kind of interventions that are relevant to RESTORE, but will provide flexibility to allow for country- and culture-specific issues as appropriate. The results of the mapping exercise in each country will be compiled into a comprehensive portfolio of guidelines and training initiatives. This will be a key resource for the case study component, to which we now turn.

### Case study: sampling and recruitment

Our potential range of key stakeholders are migrant service users, general practitioners, primary care nurses, practice managers and associated administrative staff, interpreters, cultural mediators, service planners, and policy makers. We will use purposive and maximum variation sampling to identify and recruit key stakeholder representatives in primary care settings in our local contexts. In terms of maximum variation sampling, there is diversity within our collaboration to recruit stakeholders from: urban and rural areas; areas with higher and lower densities of migrant service users; different primary care settings; expertise/interest in a range of clinical conditions; and migrants from different countries and with different legal status.

**Figure 3:**
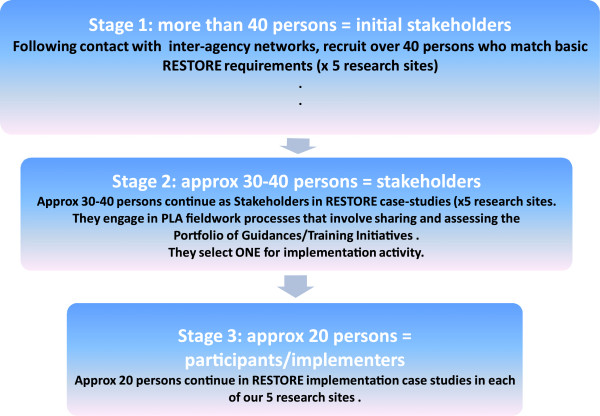
Overview of sampling and recruitment in RESTORE case study.

At the beginning of our case study (Figure 3, Stage 1), we will invite stakeholders to learn about RESTORE. Stakeholders who would like to become actively involved will then be provided with an opportunity to examine the portfolio of guidelines and training initiatives compiled from the aforementioned mapping exercise. Stakeholders will examine the portfolio to see if there is an intervention they wish to implement in their local settings (Figure 3, Stage 2). Clearly, not all stakeholders involved in this early fieldwork will want to/be able to ‘buy into’ a specific implementation project, and those who do will be making a commitment to taking on implementation work over an extended period. Therefore, through our fieldwork, it is expected that the initial group of stakeholders will ‘narrow down’ in size as stakeholders elect, or not, to become participants in a shared implementation project (Figure 3, Stage 3).

In keeping with the qualitative design, sample size for each stage will be determined throughout fieldwork by the use of theoretical saturation [54].

### Case study data generation and analysis using PLA and NPT

To generate data in Stages 1, 2, and 3 of our case study, we will engage in a PLA-brokered dialogue with stakeholders in each setting. This PLA dialogue will be conducted using interview and focus group methods. A wide range of innovative and creative PLA techniques are available to us for data generation in interviews and focus groups across our diverse stakeholder groups. These PLA techniques combine standard qualitative interviewing processes with creative visual techniques, which are ideal for use with high-, mixed- or low-level literacy groups or groups with mixed language abilities, thereby ensuring an inclusive mode of engagement across stakeholder groups, which is ideal for discussion and debate of the issues under investigation. We anticipate using several core PLA techniques alongside semi-structured interviews and focus groups in order to generate, organize, and prioritize data from stakeholders’ perspectives. For example:

1. ‘Flexible Brainstorming’ to elicit stakeholders’ diverse knowledges and experiences and to establish initial connections and relationships between emerging themes.

2. ‘Timelines’, which are useful for clarifying the context in which current experiences are taking place and relationships between experiences and their chronology.

3. ‘Card Sorts’, which enable stakeholders to generate and identify data that are meaningful to them, to sort or categorize them using emic analytical categories. This allows stakeholders to have a ‘voice’ in the thematic analysis of their own data and is an example of PLA principles in action.

4. Ranking and scoring exercises, which are ideal for prioritizing potential solutions.

5. Options Assessment and other types of matrices—correlation exercises that enable stakeholders to express or identify, for example, strong or weak interconnections between data.

In addition, observation techniques may be used to assess implementation processes in action. We may, for example, observe the organizational routines that are being developed by administrative staff in a general practice setting to support the use of a professional, paid interpreter in general practice consultations, because these are known to be complex and to have an impact on the routinization process [13,18]. We will build in piloting of all data generation strategies to ensure that they address the specific empirical issues under analysis.

**Figure 4:**
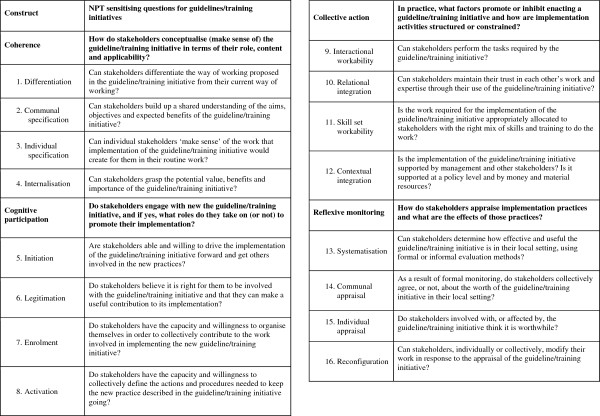
NPT sensitizing questions for RESTORE.

The purpose of our data generation is to elicit stakeholders’ views and experiences of their implementation journeys as conceptualized by the NPT. Sensitizing questions derived from the four NPT constructs—coherence, cognitive participation, collective action, and reflexive monitoring—will be used to frame data generation encounters in the PLA dialogue. These questions are shown in Figure 4 and are presented as per the temporal order of thinking about implementation work, and then actually doing implementation work. The questions are also organized neatly per construct. However, it is important to note that, in keeping with the epistemological origins of NPT and PLA and their shared emphasis on fluid and flexible realities, we expect that there will be interactions and overlaps between constructs. For example, stakeholders’ sense-making (coherence) or ‘buy in’ (cognitive participation) may change over time as they appraise the work (reflexive monitoring) that they are undertaking. This should be taken into account when reading the description of our use of these sensitizing questions below in Stages 2 and 3 of RESTORE.

As above, during Stage 2 of our case study stakeholders will examine the portfolio of guidelines and training initiatives, and we will focus our PLA dialogue on coherence*.* This NPT construct focuses on whether an intervention makes sense, or not, to relevant stakeholders and whether they see the point of it, or not, for their daily work. The empirical objective here is to determine with stakeholders how they conceptualize guidelines and training initiatives designed to support communication in cross-cultural consultations in terms of the role, content, and applicability of these guidelines and training initiatives in their everyday work settings.

The PLA-brokered dialogue will develop and merge into cognitive participation, the NPT construct that focuses on whether stakeholders actually engage or not with any of the interventions in the portfolio. This construct will encourage us to consider whether stakeholders are ready and willing to select and ‘buy into’ a specific implementation project and whether they can encourage others to do so as well. The empirical objective here is to determine with relevant stakeholders what factors promote or inhibit their engagement with potential implementation projects.

Interestingly, through this dialogue there is scope for stakeholders to co-design and fine-tune an implementation project for their specific local settings from the available portfolio. This kind of stakeholder involvement in the development of guidelines or interventions can have a positive impact on implementation processes [55] and resonates well with policy imperatives to involve service users in the development of clinical guidelines [15] and previous research [22]. Giving stakeholders a ‘voice’ in the design of their implementation project is important from a PLA perspective because co-design at the early stages of projects is a significant indicator of meaningful engagement, and this is central to good quality participatory research. It is also important from an NPT point of view because if stakeholders have a ‘voice’ in the selection or co-design, this may impact on their shared sense of the purpose and applicability of a guideline or training initiative (coherence) with which they are engaging (cognitive participation) that, in turn, may enhance the actual work involved in delivering the intervention (collective action).

Therefore, as part of our interest in the application of NPT and PLA in this field of research, we will also have the opportunity to document the specific effects of stakeholder involvement in the co-design of an intervention for their local setting on the implementation journey beyond.

As outlined earlier, during Stage 3 the sample will narrow down to a smaller group of stakeholders who commit to implementing a guideline or training initiative in their local setting over an extended period. The empirical focus here will move to collective action, which focuses on the actual work that stakeholders will undertake to implement an intervention in practice. The objective here is to determine with participants how the intervention they have bought into, and which they may have co-designed, will affect their routine work. The PLA research process will now focus clearly on participants’ experiences of implementing their chosen guideline or training initiative in practice, the documentation of any arising problems or barriers and, importantly, the co-design of potential solutions to arising problems that can be tested by participants as part of their implementation work. Taken together, these tasks create an iterative loop between analysis of initial experiences with the intervention and the work related to its implementation, exploration of potential solutions to any experienced problems, testing of identified potential solutions by participants in their implementation work, and analysis of subsequent experiences with the intervention. This process will be key to our exploration of the use of theory to identify and mediate barriers to implementation and the scope of participatory approaches to facilitate individual and organizational transformation.

This part of the PLA dialogue overlaps with the construct ‘reflexive monitoring.’ The empirical focus here is to determine with participants: how do they perceive the intervention once they have been using it for some time? For this, we will explore how participants themselves appraise the implementation they have engaged with in their local settings, and furthermore, the ways in which experiences of implementation work may shape and re-shape ‘coherence’, ‘cognitive participation’, and ‘collective action’.

Data analysis during this phase (and all phases) of the PLA-brokered dialogue will follow the principles of thematic analysis in qualitative research and will occur in iterative cycles throughout all phases of the work. We have experience of using NPT in inductive and deductive analysis [43,44], and during the iterative phases of early fieldwork we will appraise the relevance of these approaches. Importantly, where possible, preliminary analysis will involve stakeholder representatives as co-analysts; this process will enable us collectively to confirm/disconfirm initial findings, identify important leads to follow through on, and also to fine-tune the ongoing research process to ensure that all research questions are thoroughly addressed [56,57]. Layers of participatory data generation and co-analysis of data by and with key stakeholders constitute a ‘thick description’ [57] of the multi-layered reality that stakeholders experience. Overall, our data analysis will provide empirical data on the implementation journeys in each setting, but also the robustness of the NPT and PLA to explain and support the work involved.

## Discussion

We have described the focus of our research agenda, which is to conduct primary care research across five European settings to both investigate and support the implementation of guidelines or training initiatives that have been designed to support communication in cross-cultural general practice consultations. For this, we are employing a unique combination of NPT and PLA, which offers a novel approach to the analysis of implementation processes to address the five primary study objectives.

Our research design will allow us to apply NPT prospectively, to follow the experience of implementers in real space and time as they move from ‘thinking about doing’ (coherence and cognitive participation) to ‘doing the doing’ and appraising their work (collective action and reflexive monitoring). This is an important advance for NPT research, and a key question is whether this is advantageous to the implementation work in any way.

Another interesting feature of combining NPT with PLA is that while NPT and its constructs represent an etic perspective as per the pre-determined conceptual framework of NPT, the ethos and nature of a PLA dialogue is to encourage attention to emic perspectives and to ensure that these are also surfaced, made explicit and honored across stakeholder groups as valid knowledges for sharing and consideration [40]. We are, therefore, also interested in the ways in which emic perspectives may reveal ‘gaps’ in the conceptual framework of NPT and this will contribute to our testing of it, in use, in our settings.

We will be exploring the actual scope of PLA to engage and support stakeholders to identify solutions to problems as they are identified in real space and time during the implementation journey. Can the PLA dialogue enhance thinking and action about barriers, foster transformative actions and, through this process, improve the likelihood of successful implementation?

A possible threat to our case study is the potential for attrition and the loss of stakeholder engagement in the process, as we need sustained stakeholder involvement for the duration of the actual implementation work (Figure 3, Stage 3). We are also aware that the challenging and fast-changing economic climate in Europe is another potential threat [53]. We will monitor these issues carefully, and compare and contrast the emerging findings in our different settings so that we can determine shared and differential levers and barriers to normalization of the selected interventions.

Taken together, these research findings will provide an advance on state-of-the-art implementation research, because we seek to enhance implementation processes by combining a comprehensive conceptual framework of knowledge with a robust and participatory mode of action, with keen attention at all times to mediating barriers to the implementation of guidelines and training initiatives in primary care settings.

## Competing interests

AMacF, FM, COD, and CD are members of the international study group on NPT; MORdeB and TdeB are Co-Founders and Co-Directors of the Centre for Participatory Strategies, Co. Galway, Ireland.

## Authors’ contributions

AMacF is Principal Investigator and led the write-up of this paper with input from CD, FM, and COD. All authors contributed to the intellectual development of the proposal described and read and commented on drafts of this paper. All authors read and approved the final manuscript.

## Supplementary Material

Additional file 1 FP7 RESTORE Abbreviated signed GA. Click here for file

Additional file 2 FP7 RESTORE Ireland. Click here for file

Additional file 3 FP7 RESTORE England. Click here for file

Additional file 4 FP7 RESTORE the Netherlands. Click here for file

Additional file 5 FP7 RESTORE Austria. Click here for file

Additional file 6 FP7 RESTORE Greece. Click here for file
