# Supplementary material for: REsearch into implementation STrategies to support patients of different ORigins and language background in a variety of European primary care settings (RESTORE): study protocol
Source: Implement Sci. 2012 Nov 20;7:111. doi: 10.1186/1748-5908-7-111 (PMC3541149; doi:10.1186/1748-5908-7-111)
Supplement: Additional file 1 — FP7 RESTORE Abbreviated signed GA. [file 1748-5908-7-111-S1.pdf]

EUROPEAN COMMISSION

RESEARCH DIRECTORATE-GENERAL

SP1-Cooperation

Collaborative project

Small or medium-scale focused research project

FP7-HEALTH-2010-two-stage

Grant Agreement Number 257258

RESTORE

REsearch into implementation STrategies to support patients of different ORigins and language background in a variety of European primary care settings

HEALTH-F3-2010-257258

SEVENTH FRAMEWORK PROGRAMME

GRANT AGREEMENT No 257258

PROJECT TITLE RESTORE

Collaborative project

Small or medium-scale focused research project

The **European Union** ("*the Union*"), represented by the **European Commission** (the "*Commission*"),

of the **one part**,

and NATIONAL UNIVERSITY OF IRELAND, GALWAY, established in University Road -, GALWAY, ., Ireland represented by Jacinta Thornton, Commercialisation Executive and/or Neil Ferguson, Commercialisation Executive or their authorised representative, the *beneficiary* acting as "*coordinator*" of the *consortium* (the "*coordinator*"), ("*beneficiary no. 1*"),

of the **other part**

HAVE AGREED to the following terms and conditions including those in the following annexes, which form an integral part of this *grant agreement* (the "*grant agreement*").

Annex I - Description of Work
Annex II - General conditions
Annex III - Non applicable
Annex IV - Form A - Accession of *beneficiaries* to the *grant agreement*
Annex V - Form B - Request for accession of a new *beneficiary* to the *grant agreement*
Annex VI - Form C - Financial statement per funding scheme
Annex VII - Form D - Terms of reference for the certificate on the financial statements and Form E - Terms of reference for the certificate on the methodology

Article 1 - Accession to the *grant agreement* of the other *beneficiaries*

1. The *coordinator* shall endeavour to ensure that each legal entity identified below accedes to this *grant agreement* as a *beneficiary*, assuming the rights and obligations established by the *grant agreement* with effect from the date on which the *grant agreement* enters into force, by signing Form A in three originals, countersigned by the *coordinator*.

- **THE UNIVERSITY OF LIVERPOOL,** established in Brownlow Hill, Foundation Building 765 , LIVERPOOL, L69 7ZX, United Kingdom represented by Veronica Cannings, Head of Research Support or his authorised representative ("*beneficiary no. 2*"),

- **UNIVERSITY OF GLASGOW,** established in University Avenue, GLASGOW, G12 8QQ, United Kingdom represented by JOE GALLOWAY, CONTRACTS MANAGER and/or ROSS COWAN, CONTRACTS MANAGER or their authorised representative ("*beneficiary no. 3*"),

- **MEDIZINISCHE UNIVERSITAET WIEN,** established in Spitalgasse 23, WIEN, 1090, Austria represented by Wolfgang Schuetz, Rector or his authorised representative ("*beneficiary no. 4*"),

• **STICHTING KATHOLIEKE UNIVERSITEIT,** established in Comeniuslaan 4, NIJMEGEN, 6525 HP , Netherlands represented by HENDRIK-JAN VOS, DIRECTOR VALORISATION/TECHNOLOGY TRANSFER and/or FRANS CORSTENS, DEAN or their authorised representative ("*beneficiary no. 5*"),

• **PANEPISTIMIO KRITIS (UNIVERSITY OF CRETE),** established in PANEPISTIMIOUPOLI RETHIMNOU (UNIVERSITY CAMPUS, GALLOS, RETHIMNO), RETHYMNON, CRETE, 74100, Greece represented by EMMANUEL PETRAKIS, VICE RECTOR or his authorised representative ("*beneficiary no. 6*"),

• **PINTAIL LTD,** established in SPRINGHILL AVENUE 77, BLACKROCK, Ireland represented by Ciaran Clissmann, Director or his authorised representative ("*beneficiary no. 7*"),

All the *beneficiaries* together form the *consortium* (the "*consortium*").

2. The *coordinator* shall send to the *Commission* one duly completed and signed Form A per *beneficiary* at the latest 45 calendar days after the entry into force of the *grant agreement.* The two remaining signed originals shall be kept, one by the *coordinator* to be made available for consultation at the request of any *beneficiary*, and the other by the *beneficiary* concerned.

3. Should any legal entity identified above, fail or refuse to accede to the *grant agreement* within the deadline established in the previous paragraph, the *Commission* is no longer bound by its offer to the said legal entity(ies). The *consortium* may propose to the *Commission*, within the time-limit to be fixed by the latter, appropriate solutions to ensure the implementation of the *project.* The procedure established in Annex II for amendments to this *grant agreement* will apply.

Article 2 - Scope

The Union has decided to grant a financial contribution for the implementation of the *project* as specified in Annex I, called *REsearch into implementation STrategies to support patients of different ORigins and language background in a variety of European primary care settings* (*RESTORE*) (the "*project*") within the framework of the *SP1-Cooperation* and under the conditions laid down in this *grant agreement.*

Article 3 - Duration and *start date* of the *project*

The duration of the *project* shall be 48 months from 1st April 2011 (hereinafter referred to as the "*start date*").

Article 4 - Reporting periods and language of reports

The *project* is divided into reporting periods of the following duration:

• P1: from month 1 to month 18

• P2: from month 19 to month 36

• P3: from month 37 to the last month of the *project.*

Any report and deliverable, when appropriate, required by this *grant agreement* shall be in *English.*

Article 11 - Entry into force of the *grant agreement*

This *grant agreement* shall enter into force after its signature by the coordinator and the *Commission*, on the day of the last signature.

Done in two originals in English.

For the *coordinator* done at GALWAY

NATIONAL UNIVERSITY of IRELAND, GALWAY
....
Name of the legal entity

JACINTA THORNTON
....
Name of the legal representative

....
Stamp of the organisation (if applicable)

....
Signature of legal representative

17/11/10
....
Date

National University Of Ireland, Galway
17 NOV 2010
TECHNOLOGY TRANSFER OFFICE

For the *Commission* done at Brussels

Ruxandra DRAGHIA-AKLI
Director

....
Name of the legal representative

....
Signature of legal representative

31 DEC. 2010
....
Date

EUROPEAN COMMISSION
Research Directorate-General
