# Supplementary material for: REsearch into implementation STrategies to support patients of different ORigins and language background in a variety of European primary care settings (RESTORE): study protocol
Source: Implement Sci. 2012 Nov 20;7:111. doi: 10.1186/1748-5908-7-111 (PMC3541149; doi:10.1186/1748-5908-7-111)
Supplement: Additional file 3 — FP7 RESTORE England. [file 1748-5908-7-111-S3.docx]

**Monday March 19^th^ 2012 – email correspondence confirming situation re ethics in Nijmegen**

Dear Anne and Maria,

In the email below the original message in Dutch.

It says we do not need Ethical approval for this study if any questionnaires we plan to use are not to confrontational and too much of a burden for the participants. I also asked if they needed to see the questionnaires in case we will use any, , and they said: if we in any way are in doubt, then please ask the committee to have a look and decide if they might have to reconsider their opinion and perhaps approval is needed.

I hope this is enough?

Kind regards,

Evelyn

**Van:** Vlist, Maaike van der **Namens** cmo
**Verzonden:** maandag 27 december 2010 13:39
**Aan:** Weel-Baumgarten, Evelyn van
**Onderwerp:** 2010/436

## Titel: Research into implemantation strategies to support patients of different origins and language background in a variety of European primary care settings. RESTORE

**Registratienummer: 2010/436**

Geachte Dr. E. van Weel-Baumgarten,

In antwoord op uw brief van 29 november 2010 bericht ik u als volgt.

Naar het oordeel van de commissie is het voorgenomen onderzoek niet WMO-plichtig als tenminste  de vragenlijsten die gebruikt gaan worden niet belastend of confronterend zijn. Aangezien de vragenlijsten nog niet beschikbaar zijn kan de commissie daarover nu geen oordeel geven.

Met vriendelijke groet,

Dr. F.Th.M. Huysmans

**Universitair Medisch Centrum St Radboud**

**IWKV - Commissie Mensgebonden Onderzoek**

Huispost 578, route 578

Postbus 9101

6500 HB Nijmegen

Telefoon: (024) 36 13154

E-mail: [cmo@iwkv.umcn.nl](mailto:cmo@medzaken.umcn.nl)

[http://portal.umcn.nl/organisatie/iwkv](http://portal.umcn.nl/organisatie/IWKV/Pages/home.aspx)

[www.cmoregio-a-n.nl](http://www.cmoregio-a-n.nl/)

Het UMC St Radboud staat geregistreerd bij de Kamer van Koophandel in het handelsregister onder nummer 41055629.
The Radboud University Nijmegen Medical Centre is listed in the Commercial Register of the Chamber of Commerce under file number 41055629.
