# Supplementary material for: REsearch into implementation STrategies to support patients of different ORigins and language background in a variety of European primary care settings (RESTORE): study protocol
Source: Implement Sci. 2012 Nov 20;7:111. doi: 10.1186/1748-5908-7-111 (PMC3541149; doi:10.1186/1748-5908-7-111)
Supplement: Additional file 5 — FP7 RESTORE Austria. [file 1748-5908-7-111-S5.docx]

Borschkegasse 8b/6

A-1090 Wien, Austria

T +43 1 40 400 – 2147, 2244

F +43 1 40 400 – 1690 [ethik-kom@meduniwien.ac.at](mailto:ethik-kom@meduniwien.ac.at) ethikkommission.meduniwien.ac.at

**Votum:**

**EK Nr: 1081/2012**

**Project title:** Research into implementation strategies to support patients of different origins and language background in a variety of European primary care settings
**Applicant:** Dr. Wolfgang Spiegel
**Institution:** Medical University of Vienne, Centre for Public Health, Department of General Practice **Sponsor:** MedUni Wien, RESTORE-Project

Examination Centers:

| **Ethic commission** | **Examination center** | **Investigator** |
| --- | --- | --- |
| Ethic commission of the Medical University of Vienna | Medical University of Vienne, Centre for Public Health, Department of General Practice | Dr. Wolfgang Spiegel |

The statement of the ethic commission relates due to the following submitted documents:

| **Dokument** | **Name** | **Version** | **Datum** |
| --- | --- | --- | --- |
| Conflict of Interest | Conflict of Interest | 1.1. | 02.01.2012 |
| Lebenslauf (CV) | CV_Spiegel | 1.1. | 08.02.2012 |
| Others | Ethikkommissionsvotum England | 1.1. | 25.03.2011 |
|  | Ethikkommissionsvotum Irland | 1.1. | 01.12.2010 |
|  | Ethikkomissionsvotum Griechenland | 1.1. | 20.09.2010 |
|  | Ethikkommissions Griechenland (englische Übersetzung) | 1.1. | 20.09.2010 |
| Patient information | TeilnehmerInneninformation_für_restliche_TeilnehmerInnen_de_2012-  06-27_v1.3 | 1.3. | 27.06.2012 |
|  | TeilnehmerInneninformation_für_MigrantInnen_de_2012-06-19_v2.3. | 2.3. | 19.06.2012 |
| Clinical study protocol | Clinical Study Protocol_2012-05-03_v1.2 | 1.2. | 03.05.2012 |

**The ethic commission makes the following decision (signed with an X):**

**X** There is no reason to object the conducting of the study.

**Comments:**

About the clinical study protocol:

Obviously a not applicable template was filled in for the clinical study protocol (clinical study protocol – Austrian Medicine Law). As example there are points about the application of GCP, serious adverse events etc. Because there is no clinical trial, the study protocol has to be adapted.

About the participant information:
It should be filled in, which kind of person groups will participate in this study.
It should be filled in, how long the participant will have to participate in this study.
The details of a „focus group „should be more described. Within the application, it is mentioned that “if any participant has physical and/or psychological strains – during the study phase – we will have the possibility to refer them to an appropriate source of our professional health care network.” This also must be mentioned within the participant information (point 4, risks). The text of the participant information is written a little bit too detailed and complicated (“reduction of language and cultural barriers”, “participation on focus group”, “primary health care consultations”) for migrant service users. There has to be a separately worded version for migrant service users

The ethic commission requests from the applicant a version of the participant information with highlighted changes.
About the insurance confirmation: not necessary

The ethic commission does not expect that this clinical study underlies the Austrian Medical Law/Austrian Medical Product Law.

**Additional comments:**

Addendum from the 28th June 2012: The applicants present adapted documents on the 28.06.2012, which are accepted by the ethics commission.

The ethic commission does not expect, that this clinical study underlies the Austrian Medical Law/Austrian Medical Product Law.
You can find the current member list of the ethics comission under the following address: <http://ethikkommission.meduniwien.ac.at/ethik-kommission/mitglieder/>. Members, which were biased under this agenda, did not participate in the vote/decision-making: Dr. med. Kathryn Hoffmann, Mr. Thomas Waldhör

**ATTENTION:** Regarding the "ICH-Guideline for Good Clinical Practice" this vote is valid for **one year as of the date of its issuance.** If necessary the applicant has to apply an extension of the validation in time.

Page 2/2
