# Supplementary material for: REsearch into implementation STrategies to support patients of different ORigins and language background in a variety of European primary care settings (RESTORE): study protocol
Source: Implement Sci. 2012 Nov 20;7:111. doi: 10.1186/1748-5908-7-111 (PMC3541149; doi:10.1186/1748-5908-7-111)
Supplement: Additional file 6 — FP7 RESTORE Greece. [file 1748-5908-7-111-S6.doc]

**
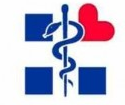
**

**MINISTRY OF HEALTH AND WELFARE**

**HEALTHCARE MANAGEMENT REGION OF CRETE**

**UNIVERSITY GENERAL HOSPITAL HRAKLEIO**

**P.O. Box 1352 Voutes, Hrakleio Crete**

*Translation from*

*the original document*

**SCIENTIFIC COUNCIL**

Heraklion 20/9/2010

Protocol No: 8297

**PRESIDENT**

Dimitris Georgopoulos

Professor

Director

**ASSOCIATE PRESIDENT**

Marioris Andreas

Director Chemistry Biochemistry Lab

**MEMBERS**

Venoxaki Maria

Assist. Professor -Clinic of Chemistry & Biochemistry

Georgios Maltezakis

Director National Health System

Kalapakis Konstantinos

General surgeon

Mathiodakis Emmanouel

Anesthesiologist

Prinari Aggeliki

Nurse Practitioner

Mandolas Panagiotis

Medical lab

Secretary

Maria Klinaki

Tel: 2810392478

Fax:2810392478

TO: Director Bio-Ethic Committee

Prof. A. Mariori

**SUBJECT: Approval of research protocol that will be held at the Clinic of Social and Family Medicine at the University Hospital Pepagni**

The Scientific Council at the 9/9/2010 meeting taking into account Prof. Christos Lionis’ proposal with research number 8297/19-7-2010 expresses the positive recommendation from the working group of Bio-Ethical Committee of the supporting documents sent therefore approving the research protocol entitled “Research into implantation strategies to support patients of different origins and language background in a variety of European primary care settings” in which will be implemented by the Clinic of Social and Family Medicine of the University Hospital.

The Scientific Council

Professor Dimitris Georgopoulos
