# Supplementary material for: REsearch into implementation STrategies to support patients of different ORigins and language background in a variety of European primary care settings (RESTORE): study protocol
Source: Implement Sci. 2012 Nov 20;7:111. doi: 10.1186/1748-5908-7-111 (PMC3541149; doi:10.1186/1748-5908-7-111)
Supplement: Additional file 2 — FP7 RESTORE Ireland. [file 1748-5908-7-111-S2.pdf]

The Irish College of General Practitioners

Coláiste Dhochtúirí Teaghlaigh Éireann

13th July 2012

Prof Anne MacFarlane
Professor of Primary Healthcare Research
Graduate Entry Medical School,
University of Limerick
Limerick

RESTORE project

Dear Prof McFarlane

Thank you for the further information regarding the RESTORE project.

We are happy to approve the data protection arrangements as per your reply.

We welcome the further information regarding the recruitment process but would still be anxious to know which stakeholder groups, particularly of services users, are actually recruited from in the end. You mention Galway Refugee Support Group and Galway Migrant Group as examples. Please keep us informed if you recruit from these and/or other groups.

The use of interpreters and their availability should be made clear to participants. You say such support will be provided 'if necessary' but we are still unclear as to how this necessity will be decided. In our view the necessity for interpreters should be determined by service users and not be left to the judgement of the researchers. Thus, it would be our preference that interpretation services be offered to all non-native English speaking participants automatically and then whether or not such services are availed of is at their decision rather than having to be judged/decided by researchers.

We note the PIL will be translated in to different languages for non-native English speakers

We accept your comments regarding the number of meetings but are pleased to see the frequency of every 6-8 weeks notified in the information sheet.

In view of these replies we are happy to approve the project subject to the following two additional requirements:

1) We are kept informed of the exact stakeholder groups from whom recruitment is undertaken
2) Non native English speaking participants are offered interpretation services by default rather than 'if necessary'. Necessity for such services to be determined by research participants

Yours Sincerely

Prof Colin Bradley
In my capacity as Chair of ICGP Research Ethics Committee

4-5 Lincoln Place, Dublin 2
Tel: (01) 676 3705/06 Fax: (01) 676 5850
e-mail: info@icgp.ie www.icgp.ie

Regd. in Ireland at the Irish College of General Practitioners Limited
Registration Number 100456

Kieran Ryan
Chief Executive Officer
