# Supplementary material for: REsearch into implementation STrategies to support patients of different ORigins and language background in a variety of European primary care settings (RESTORE): study protocol
Source: Implement Sci. 2012 Nov 20;7:111. doi: 10.1186/1748-5908-7-111 (PMC3541149; doi:10.1186/1748-5908-7-111)
Supplement: Additional file 4 — FP7 RESTORE the Netherlands. [file 1748-5908-7-111-S4.pdf]

NHS
National Research Ethics Service
North West 9 Research Ethics Committee – Greater Manchester West
Barlow House
3rd Floor
4 Minshull Street
Manchester
M1 3DZ

Telephone: 0161 625 7821
Facsimile:

25 March 2011

Professor Christopher Dowrick
Professor of Primary Medical Care
University of Liverpool
1st Floor Waterhouse Building B
1-5 Brownlow Street
Liverpool
L69 3GL

Dear Professor Dowrick

| | |
|---|---|
| **Study title:** | **Research into implementation strategies to support patients of different originsand language background in a variety of European primary care settings** |
| **REC reference:** | **11/NW/0052** |
| **Protocol number:** | **UoL0000671** |

Thank you for your letter of 22 March 2011, responding to the Committee's request for further information on the above research and submitting revised documentation.

The further information has been considered on behalf of the Committee by the Chair.

**Confirmation of ethical opinion**

On behalf of the Committee, I am pleased to confirm a favourable ethical opinion for the above research on the basis described in the application form, protocol and supporting documentation as revised, subject to the conditions specified below.

**Ethical review of research sites**

NHS sites

The favourable opinion applies to all NHS sites taking part in the study, subject to management permission being obtained from the NHS/HSC R&D office prior to the start of the study (see "Conditions of the favourable opinion" below).

**Conditions of the favourable opinion**

The favourable opinion is subject to the following conditions being met prior to the start of the study.

Management permission or approval must be obtained from each host organisation prior to the start of the study at the site concerned.

*Management permission ("R&D approval") should be sought from all NHS organisations involved in the study in accordance with NHS research governance arrangements.*

Guidance on applying for NHS permission for research is available in the Integrated Research Application System or at http://www.rdforum.nhs.uk.

*Where a NHS organisation's role in the study is limited to identifying and referring potential participants to research sites ("participant identification centre"), guidance should be sought from the R&D office on the information it requires to give permission for this activity.*

*For non-NHS sites, site management permission should be obtained in accordance with the procedures of the relevant host organisation.*

*Sponsors are not required to notify the Committee of approvals from host organisations*

**It is the responsibility of the sponsor to ensure that all the conditions are complied with before the start of the study or its initiation at a particular site (as applicable).**

**Approved documents**

The final list of documents reviewed and approved by the Committee is as follows:

| *Document* | *Version* | *Date* |
|---|---|---|
| Protocol | 1 | 15 September 2010 |
| Letter of invitation to participant | 1 | 16 November 2010 |
| Response to Request for Further Information | | 22 March 2011 |
| Participant Information Sheet: Service Provider | 3 | 22 March 2011 |
| REC application | 3.1 | 24 January 2011 |
| Copy of Unfavorable Opinion Letter | | 14 January 2011 |
| Participant Information Sheet: Service User | 3 | 22 March 2011 |
| Evidence of insurance or indemnity | Clinical Trials Compensation Insurance (CLIN97UK06AN) | 03 August 2010 |
| Referees or other scientific critique report | RESTORE: Response to Evaluator's Comments | 25 May 2010 |
| Referees or other scientific critique report | External Assessment on Sampling Strategy | 30 January 2011 |
| Investigator CV | Christopher Dowrick | |
| Participant Consent Form | 3 | 22 March 2011 |
| Covering Letter | | 02 February 2011 |
| Letter from Sponsor | | 26 November 2010 |

**Statement of compliance**

The Committee is constituted in accordance with the Governance Arrangements for Research Ethics Committees (July 2001) and complies fully with the Standard Operating Procedures for Research Ethics Committees in the UK.

**After ethical review**

Now that you have completed the application process please visit the National Research Ethics Service website > After Review

You are invited to give your view of the service that you have received from the National Research Ethics Service and the application procedure. If you wish to make your views known please use the feedback form available on the website.

The attached document *"After ethical review – guidance for researchers"* gives detailed guidance on reporting requirements for studies with a favourable opinion, including:

- Notifying substantial amendments
- Adding new sites and investigators
- Progress and safety reports
- Notifying the end of the study

The NRES website also provides guidance on these topics, which is updated in the light of changes in reporting requirements or procedures.

We would also like to inform you that we consult regularly with stakeholders to improve our service. If you would like to join our Reference Group please email referencegroup@nres.npsa.nhs.uk.

| **11/NW/0052** | **Please quote this number on all correspondence** |
|---|---|

With the Committee's best wishes for the success of this project

Yours sincerely

pp

**Dr Lorraine Lighton**
**Chair**

Email: Shehnaz.ishaq@northwest.nhs.uk

Enclosures: "After ethical review – guidance for researchers"

Copy to:

Maxine Martin
2nd Floor Waterhouse Buildings B
1-5 Brownlow Street
Liverpool
L69 3GL

Gabrielle Marr
Liverpool PCT
1 Arthouse Square, 3rd Floor
61-69 Seel Street
Liverpool
L1 4AZ

Lindsay Carter - Research Co-ordinator, University of Liverpool
